# Comparative Study of Gleason 7 (3+4) and (4+3) Prostatic Adenocarcinomas with Prognostic Criteria and Immunohistochemical Profiles of AMACR, PSA and Ki-67

**DOI:** 10.1590/S1677-5538.IBJU.2024.9922

**Published:** 2025-01-10

**Authors:** Clarice F. E. M. Osório, Waldemar S. Costa, Carla B. M. Gallo, Luciano A. Favorito, Francisco J. B. Sampaio

**Affiliations:** 1 Universidade do Estado do Rio de Janeiro Unidade de Pesquisa Urogenital Rio de Janeiro RJ Brasil Unidade de Pesquisa Urogenital – Universidade do Estado do Rio de Janeiro – UERJ, Rio de Janeiro, RJ, Brasil

**Keywords:** Prostatic Neoplasms, Neoplasm Grading, Prognosis, Histological Techniques

## Abstract

**Background:**

To compare Gleason 7 (3+4) and (4+3) prostatic adenocarcinoma (PC) with different prognostic criteria through immunohistochemical analysis with anti-PSA, anti-Ki 67 and anti-AMARC antibodies.

**Methods:**

We analyzed 221 surgical specimens from patients between 40 and 86 years-old (mean=63) with PC. The immunohistochemical study was performed with anti-PSA, anti-Ki 67 and anti-AMARC. The microscopic fields were photographed with an Olympus DP70 digital camera coupled to an Olympus BX51 microscope and archived in TIFF. Proportion and intensity criteria were used to quantify the anti-PSA antibody and for the anti-Ki 67 antibody, the quantification by similarity of this antibody in breast carcinomas. Anti-AMACR protein expression was based on four scores: negative, weak, moderate and strong. The statistical analysis was performed with the Graph Pad Prism 5 program.

**Results:**

In the Gleason score 7 (3+4) we had 91.72% in pT2 and 8.27% in the pT3 group; 8.27% recurrences, of which 90.90% in the pT2 group. In the Gleason score 7 (4+3) we had 77.27% in the pT2 group and 22.72% in the pT3 group and 10.22% of relapses, of which 66.66% in the pT2 group and 33.33% in the pT3 group. In 6.81% of cases there was an increase in the anti-Ki 67 index and in 2.27% of the cases, there was an increase in the immunoexpression of anti-p53 when comparing Gleason score 7 (3+4) with Gleason score 7 (4+3).

**Conclusion:**

Our study confirmed differences in the Gleason score 7 (3+4) and Gleason score 7 (4+3) of PC when comparing prognostic criteria. Anti-Ki 67 and anti-PSA antibody immunostaining showed a positive correlation as the Gleason score 7 increased from (3+4) to (4+3).

## INTRODUCTION

The classification of prostatic adenocarcinomas is still based on Gleason's histopathological classification, which, despite having undergone modifications, remains a well-established indicator that has supported the test of time (1, 2). In 2013, a new system composed of grade groups of 1 to 5 was developed by the Johns Hopkins Hospital and validated in a large multi-institutional and multimodal study by the International Society of Urological Pathology (ISUP) in 2014 (3, 4).

The new grading system proves to be more accurate than the Gleason system. The Gleason system with its primary and secondary patterns is a subjective and complicated classification system, considering that the classification systems used for other tumors are usually simplified, ranging from grade 1 to 3 (well, moderately and poorly differentiated) or low, intermediate or high grade. The current classification proposed by ISUP and WHO (World Health Organization) uses a grade group 1 for Gleason 6 and a grade group 2 for Gleason (3+4), making common prognostic and therapeutic proposals possible (3, 4). Similarly, classification in grade group 4 for Gleason 8 and grade group 5 for Gleason 9 or 10 allows for better stratification and future studies to determine whether PC grade group 5 will require more intensive therapy (3, 4).

In addition to the histopathological grading, the set of factors that determine the pathological staging is of prognostic importance, such as the invasion of the prostatic capsule, the impairment of the surgical resection margin, the impairment of the urethral and vesical margins and the invasion of seminal vesicles (5–7).

Currently, the established prognostic factors for prostate cancer (PC) are the TNM staging of the tumor (primary tumor, regional lymph nodes and distant metastases) of the AJCC (American Joint Committee on Cancer) (8), status of surgical margins, level of serum PSA and Gleason score (2).

The aim of this study was to compare Gleason 7 (3+4) and Gleason 7 (4+3) adenocarcinomas with different prognostic criteria: impairment of the surgical margin of resection, impairment of the vesical margin, impairment of the urethral margin and impairment of seminal vesicles through an immunohistochemical analysis with anti-PSA, anti-Ki 67 and anti-AMARC antibodies.

## MATERIAL AND METHODS

The project was submitted and approved by the Ethics Committee (CAAE number 12685413.6.0000.5259 of the State University of Rio de Janeiro) and is in accordance with the Institution's ethical standards for experiments with human materials.

We retrospectively analyzed 221 surgical specimens of patients (aged 40 to 86, mean=63 years old) with diagnosis of prostatic adenocarcinoma submitted to radical prostatectomy between January 2015 and January 2021.

As inclusion criteria, only cases with histopathological diagnosis of prostatic acinar adenocarcinoma Gleason 7 (3+4) and Gleason 7 (4+3) were considered. As exclusion criteria were considered the ductal pattern adenocarcinomas, other histopathological variants of prostate cancer and prostate adenocarcinomas with histopathological evidence of previous treatment.

The specimens were sent to the Pathological Anatomy Laboratory, fixed in block by immersion in 10% buffered formalin for 24 hours. The surgical margins were marked with India ink and the prostate was separated into two lobes, right and left, and respective anterior and posterior quadrants, from which samples were taken for histological analysis. Also, fragments of the vesical (upper) and urethral (lower) limits were removed.

The removed fragments were accommodated in plastic cassettes and processed for inclusion in paraffin. The samples were dehydrated in increasing baths of ethanol, diaphanized in xylene and impregnated in liquid paraffin with subsequent hardening at room temperature.

### Histologic techniques

After routinely processed for paraffin embedding, 5-μm thick sections are obtained at 200-μm intervals. Sections are stained with hematoxylin-eosin to assess the integrity of the tissue.

The immunohistochemical analysis of the prostate fragments is done with anti-PSA (prostate-specific antigen, clone EP109, Biocare), anti-Ki 67 (Molecular immunology Borstel 1, Clone MIB-1, Agilent) and anti-AMARC (alpha-methylacyl-coenzyme A racemase, P504s, clone 13H4, Cell score). The antibodies are revealed with the use of a Mouse Rapid Staining Kit (Sto Stock #1, Quik-1 - Sigma Chemical Co., St. Louis, USA), containing a secondary antibody, peroxidase, 3% hydrogen peroxide and the chromogen AEC (3-amino-9-ethyl-carbazole).

Anti-PSA is a protease of the kallikrein family produced by the ductal epithelium and prostatic acinar. Anti-PSA antibody exhibits cytoplasmic labeling in more than 95% of prostatic carcinomas, but its immunoreaction decreases as the Gleason grade increases (9).

Anti-Ki 67 is a monoclonal antigen that interacts with the human nuclear Ki 67 antigen, present in all active phases of the cell cycle (phases G1, S, G2 and M), not being present only in phase G0. Anti-Ki 67 antibody labeling is therefore nuclear and expresses cell proliferation, useful in mitotic counting (10).

Anti-AMACR is a mitochondrial and peroxisomal enzyme involved in the metabolism of branched chain of fatty acids and bile acid (11).

### Quantification Techniques

The microscopic fields were photographed, under the same conditions and by the same senior pathologist, using an Olympus DP70 digital camera (Olympus America, Inc., Melville, New York) with a resolution of 2,040 1,536 pixels, directly attached to an Olympus BX51 microscope and filed in TIFF.

The criterion for interpreting the protein expression of anti-AMACR included four scores: negative, weak (weak or apical granular cytoplasmic staining), moderate (diffuse granular cytoplasmic staining), and strong (diffuse intense cytoplasmic staining). Only moderate and strong markings were considered positive (12).

Proportion and intensity criteria were used to quantify the anti-PSA antibody based on the quantification by similarity of this antibody in hormone receptors (estrogen and progesterone) (13).

The criteria for interpretation of the anti-PSA immunostaining in patterns 3 and 4 prostate adenocarcinomas were as follows:

– Proportion: 0: unmarked; 1: less than 1%; 2: between 1 and 10%; 3: between 10% to 33%; 4: between 33 to 66%; 5: above 66%.
– Intensity: 0: no marking; 1: light; 2: moderate; 3: intense.
– Total score: sum up to 2: negative; sum greater than 2: positive. (13) (Table-1).

**Table 1:** Quantification of anti-AMARC, anti-PSA and anti-Ki67 antibodies in prostatic adenocarcinomas.

GLEASON	AMARC	PSA	KI 67
Pacient 1- Gl 3	weak (-)	P5 + I 3= 8 (+)	< 14%
Pacient 1 - Gl 4	weak (-)	P5 + I 3= 8 (+)	> 14%
Pacient 2 - Gl 3	weak (-)	P 5 + I 3= 8(+)	< 14%
Pacient 2 - Gl 4	moderate (+)	P5 + I 3= 8 (+)	< 14%
Pacient 3 - Gl 3	weak (-)	P 5 + I 2= 7 (+)	< 14%
Pacient 3 - Gl 4	weak (-)	P 5 + I 3= 8 (+)	14%
Pacient 4 - Gl 3	weak (-)	P 4 + I 2= 6 (+)	< 14%
Pacient 4 - Gl 4	weak (-)	P5 + I 3= 8 (+)	14%
Pacient 5 - Gl 3	moderate (+)	P 5 + I 2= 7 (+)	< 14%
Pacient 5 - Gl 4	moderate (+)	P5 + I 3= 8 (+)	< 14%
Pacient 6 - Gl 3	weak (-)	P 5 + I 1= 6 (+)	< 14%
Pacient 6 - Gl 4	weak (-)	P 5 + I 2= 7 (+)	14%
Pacient 7 - Gl 3	weak (-)	P 5 + I 3= 8 (+)	< 14%
Pacient 7 - Gl 4	weak (-)	P5 + I 3= 8 (+)	< 14%
Pacient 8 - Gl 3	moderate (+)	P 5 + I 2= 7 (+)	14%
Pacient 8 - Gl 4	moderate (+)	P 5 + I 1= 6 (+)	14%
Pacient 9 - Gl 3	weak (-)	P 5 + I 2= 7 (+)	< 14%
Pacient 9 - Gl 4	moderate (+)	P5 + I 3= 8 (+)	> 14%
Pacient 10 - Gl 3	weak (-)	P 2 + I 1= 3 (+)	< 14%
Pacient 10 - Gl 4	weak (-)	P3 + I 2= 5 (+)	< 14%
Pacient 11 - Gl 3	weak (-)	P 5 + I 3= 8 (+)	< 14%
Pacient 11 - Gl 4	weak (-)	P 5 + I 3= 8 (+)	< 14%
Pacient 12 - Gl 3	weak (-)	P 5 + I 3= 8 (+)	< 14%
Pacient 12 - Gl 4	weak (-)	P5 + I 2= 7 (+)	> 14%
Pacient 13 - Gl 3	weak (-)	P 5 + I 3= 8(+)	< 14%
Pacient 13 - Gl 4	weak (-)	P5 + I 3= 8 (+)	< 14%
Pacient 14 - Gl 3	moderate (+)	P 5 + I 3= 8(+)	< 14%
Pacient 14 - Gl 4	weak (-)	P5 + I 3= 8 (+)	< 14%
Pacient 15 - Gl 3	weak (-)	P3 + I 2= 5 (+)	< 14%
Pacient 15 - Gl 4	weak (-)	P3 + I 2= 5 (+)	< 14%

The anti-Ki 67 antibody quantification criterion was used based on the similarity quantification of this antibody in breast carcinomas (14).

The criterion for interpretation of the anti-Ki 67 immunostaining in prostate adenocarcinomas with Gleason patterns 3 and 4 used was the cutoff point of 14% (14).

### Statistical analysis

All parameters were statistically processed and tabulated. The Student t-test was used for comparison of quantitative data between negative vs. positive result (p<0.05). The chi-square test was used to verify associations between categorical variables and negative vs. positive result (p<0.05). The statistical analysis was performed with the Graph Pad Prism 5 program (Version 5).

## RESULTS

In the Table-2 we can observe the AJCC Pathological Classification of Gleason 7 (3+4) and Gleason 7(4+3) prostatic adenocarcinomas and the demographic data of our sample.

**Table 2:** The table shows the AJCC Pathological Classification of Gleason 7 (3+4) [A] and Gleason 7 (4+3) [B] prostatic adenocarcinoma.

	Cases		%		Age		Recurrence		White		Brown		Black	
	[A]	[B]	[A]	[B]	[A]	[B]	[A]	[B]	[A]	[B]	[A]	[B]	[A]	[B]
pT2	74	51	55.63	57.95	40-86	51-82	7	4	50	31	10	4	3	0
pT2 R1	48	17	36.09	19.31	46-77	55-71	3	2	29	8	3	4	0	0
pT3a	1	3	0.75	3.40	76	52-69	0	0	1	2	0	1	0	0
pT3a R1	6	6	4.51	6.81	54-75	56-71	1	2	3	5	1	0	1	0
pT3b	2	5	1.50	5.68	54-66	57-76	0	1	1	4	0	1	0	0
pT3b R1	2	6	1.50	6.81	61-70	59-76	0	0	1	4	0	0	0	0

[A] Gleason 7 (3+4); [B] Gleason 7 (4+3)

In the Gleason 7 score (3+4) we had 55.63% of patients distributed in pT2 and 36.09% in pT2R1 according to the AJCC classification, that is, 91.72% of patients in pT2. In this Gleason 7 score (3+4) we had only 8.26% of patients in T3.

In the Gleason 7 score (4+3) we had 57.95% of patients distributed in pT2 and 19.31% in pT2R1, that is, 77.26% of patients in pT2 and 22.70% in T3. In this Gleason score 7(4+3), pT3 was more expressive when compared to the pT2 group.

As for recurrences in the Gleason score 7 (3+4), we had a total of 11 cases (8.27%), of which 90.90% in pT2 of the AJCC and only 9.09% in pT3. In the Gleason score 7 (4 +3) we had a total of 9 recurrences (10.22%), of which 66.66% in pT2 and 33.33% in pT3. In this Gleason score 7(4+3), pT3 values were higher when compared to pT2.

As for the color of the patients, both Gleason 7 (3+4) and (4+3) scores showed a predominance of the white race, 82.52% and 84.37%, respectively. In white individuals, both in the Gleason scores 7 (3+4) and (4+3), were distributed in pT2 in the percentages of 58.82% and 57.40%, respectively.

In the Gleason score 7 (3+4) the browns were distributed in pT2 in the percentage of 71.42% and blacks were distributed in pT2 in 75% of cases and in pT3aR1 in 25%.

In the Gleason score 7 (4+3) the browns were distributed in pT2 and pT2R1 in the percentage of 40% for both.

In 6.81% of the cases there was an increase in the anti-Ki 67 index and in 2.27% of the cases there was an increase in the immunoexpression of anti-p53 when comparing Gleason score 7 (3+4) areas with Gleason score 7 (4+3) areas.

**Figure 1:**
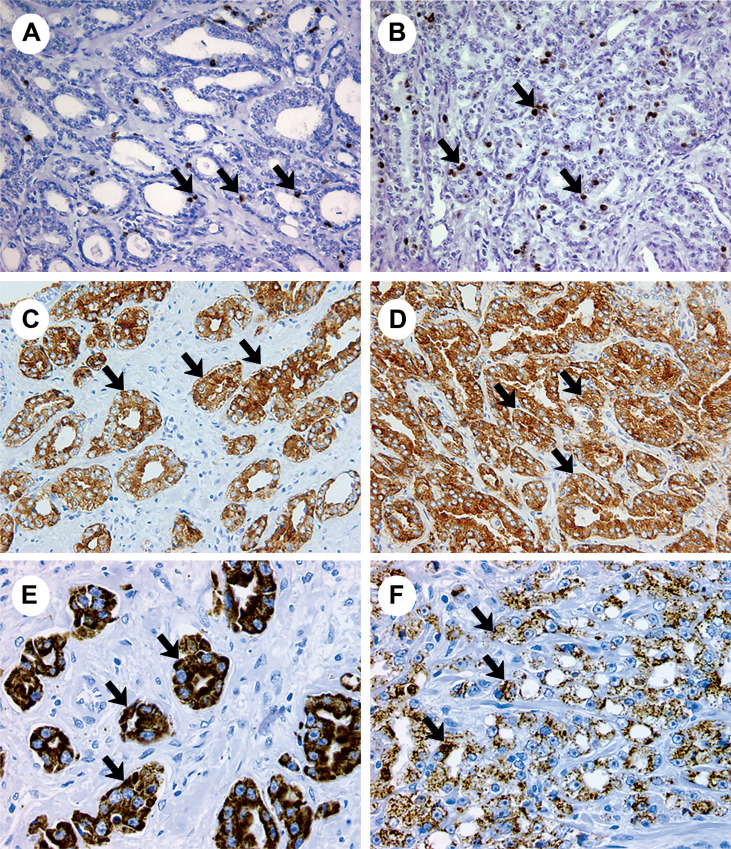
The Figure show the immunoexpression for Ki 67, PSA and AMARC in prostatic adenocarcinoma. A) Photomicrograph of anti-Ki 67 antibody (arrow) in Gleason 3 prostate adenocarcinoma (X200); B) Photomicrograph of anti-Ki 67 antibody (arrow) in Gleason 4 prostate adenocarcinoma (X200); C) Photomicrograph of immunoexpression for anti-PSA antibody (arrow) in Gleason 3 prostate adenocarcinoma (X200); D) Photomicrograph of anti-PSA antibody in Gleason 4 prostate adenocarcinoma (X400); E) Photomicrograph of anti-AMARC antibody (arrow) in Gleason 3 prostate adenocarcinoma (X200) and F) Photomicrograph of anti-AMARC antibody (arrow) in Gleason 4 prostate adenocarcinoma (X200).

In our study, 6.81% of cases showed increased immunostaining of tissue anti-PSA when compared to Gleason score 7 (3+4) with 7 (4+3) and only 2.27% of cases showed decreased PSA labeling when compared these two scores (Figure-1).

We did not find a correlation between anti-AMARC and anti-Ki 67 immunostaining.

## DISCUSSION

The AJCC methodology uses the TNM classification (T-tumor extension, N-node involvement and M-presence or absence of metastases) to group patients. TNM staging in combination with tumor grade and PSA are considered standard for PC and used in the therapeutic decision (15).

The histological grade of PC is an important, if not the most important, factor in the prognosis of clinically localized PC. For over forty years the Gleason classification was the standard classification used in the CP, based on its primary and secondary patterns from 1 to 5 and scores resulting from the sum of these patterns. In 2014 WHO and ISUP formally changed the Gleason classification and adopted a group grade system for CP. Such classification ranges from 1 to 5, where group 1 is similar to Gleason score 6, grade 2 group similar to Gleason score 7 (3+4), grade 3 group is similar to Gleason score 7 (4+3), grade 4 group is similar to Gleason score 8, and grade 5 group is similar to Gleason score 9 and 10. The eighth edition of the AJCC uses this classification (15).

The Gleason score 7 is a heterogeneous group both morphologically and in its biological behavior, so the Gleason score 7 (3+4), grade group 2, has a much more favorable prognosis than the 7 (4+3) grade group 3. This division of Gleason 7 scores implies different therapeutic strategies, especially in relation to radiotherapy (15).

Black patients have a worse prognosis for PC, related to dietary, nutritional and health factors (16, 17), although, in our results, 75% of blacks are distributed in the Gleason score 7 (3+4) pT2.

As for the color of the patients, both Gleason scores 7 (3+4) and (4+3) showed a predominance of the white race, 82.52% and 84.37%, respectively.

Prognostic factors are divided into clinical and biological. Clinical factors include PSA dosage, imaging findings, and evaluation of prostate biopsies. Biological factors are another category of prognostic factors. With recent advances in molecular biology the concept of oncogene and tumor suppressor genes are dominating tumorigenesis research and may provide new tumor markers, with special attention to anti-p53 and anti-Ki 67 (18). Verma et al., 2015 (18) in their article showed a strong relationship between the expression of anti-p53, a tumor suppressor protein, and anti-Ki 67, a cell proliferation marker, with the increase in the Gleason score, which is important for the prognosis of PC (19). Anti-p53 is an antibody homologous to anti-p63 and in benign lesions it shows discontinuous nuclear immunostaining (20).

According to Tretiakova et al. 2016 (21) the anti-Ki 67 index increases in patients with pT3/pT4 stages of the disease when compared to pT2 stages. Our results were similar, although we did not have cases of pT4 stage in these scores. Our work corroborates these results. In 6.81% of the cases there was an increase in the anti-Ki 67 index and in 2.27% of the cases there was an increase in the immunoexpression of anti-p53 when comparing Gleason score 7 (3+4) areas with Gleason score 7 (4+3) areas.

Recently, anti-AMARC overexpression has been demonstrated in localized and metastatic PC, as well as in high grade prostatic intraepithelial neoplasms, but not in normal glands, suggesting that it may be an important tumor marker (22).

Anti-PSA antibody has decreased sensitivity in poorly differentiated prostatic carcinomas, so it should be decreased in Gleason pattern 4 adenocarcinomas when compared to pattern 3 (23). In the work of Missaoui et al. 2016 (24) 78% of PCs showed anti-PSA expression. Cytoplasmic expression in tumor cells ranged from 30 to 100%.

In our study, 6.81% of the cases showed increased immunostaining of tissue anti-PSA when comparing the groups of Gleason score 7 (3+4) with that of Gleason 7 (4+3) and only 2.27% of the cases showed a decrease in labeling of PSA when comparing these two groups.

## CONCLUSIONS

Our work confirmed differences in the Gleason 7 (3+4) and Gleason 7 (4+3) scores of prostatic carcinomas when compared to the AJCC/TNM prognostic criteria.

These differences are clearer when the groups are separated only into pT2 and pT3 of the AJCC/TNM classification.

These differences corroborate with the difference in biochemical recurrence/disease-free survival after radical prostatectomy according to ISUP/WHO grade 2 and 3 groups.

Anti-Ki 67 and anti-PSA antibody immunostaining showed a positive correlation as the Gleason score 7 increased from 3+4 to 4+3.
